# Microsporidian diversity in the aquatic isopod *Asellus aquaticus*

**DOI:** 10.1017/S003118202200124X

**Published:** 2022-11

**Authors:** Daniel Grabner, Annemie Doliwa, Lidia Sworobowicz, Anna Wysocka, Alexander Weigand, Michał Grabowski, Tomasz Mamos, Bernd Sures

**Affiliations:** 1 Aquatic Ecology, University of Duisburg-Essen, Universitätsstraße 5, D-45141 Essen, Germany; 2 Centre for Water and Environmental Research, University of Duisburg-Essen, Universitätsstraße 5, D-45141 Essen, Germany; 3 Department of Evolutionary Genetics and Biosystematics, Faculty of Biology, University of Gdańsk, Wita Stwosza 59, 80-308 Gdańsk, Poland; 4 National Museum of Natural History Luxembourg, 25 Rue Munster, 2160 Luxembourg, Luxembourg; 5 Department of Invertebrate Zoology & Hydrobiology, Faculty of Biology & Environmental Protection, University of Lodz, 90-237 Łódź, Poland; 6 Water Research Group, Unit for Environmental Sciences and Management, North-West University, 11 Hoffman St, Potchefstroom, 2520, South Africa

**Keywords:** Aquatic parasites, barcoding, microsporidia, parasite diversity

## Abstract

We conducted a molecular survey on microsporidian diversity in different lineages (operational taxonomic units = OTUs) of *Asellus aquaticus* from 30 sites throughout Europe. Host body length was determined, and DNA was extracted from host tissue excluding the intestine and amplified by microsporidian-specific primers. In total, 247 *A. aquaticus* specimens were analysed from which 26.7% were PCR-positive for microsporidians, with significantly more infections in larger individuals. Prevalence ranged between 10 and 90%. At 9 sites, no microsporidians were detected. A significant relationship was found between the frequency of infected individuals and habitat type, as well as host OTU. The lowest proportion of infected individuals was detected in spring-habitats (8.7%, *n* = 46) and the highest in ponds (37.7%, *n* = 53). Proportion of infected individuals among host OTUs A, D and J was 31.7, 21.7 and 32.1%, respectively. No infections were detected in OTU F. Our results are, however, accompanied by a partially low sample size, as only a minimum of 5 individuals was available at a few locations. Overall, 17 different microsporidian molecular taxonomic units (MICMOTUs) were distinguished with 5 abundant isolates (found in 4–17 host individuals) while the remaining 12 MICMOTUs were “rare” and found only in 1–3 host individuals. No obvious spatio-genetic pattern could be observed. The MICMOTUs predominantly belonged to Nosematida and Enterocytozoonida. The present study shows that microsporidians in *A. aquaticus* are abundant and diverse but do not show obvious patterns related to host genetic lineages or geography.

## Graphical abstract

**Figure:**
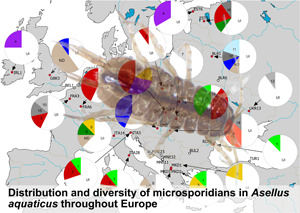

## Introduction

Microsporidians are intracellular microparasites related to the kingdom Fungi (Hirt *et al*., 1999; Capella-Gutiérrez *et al*., 2012). They infect a wide range of hosts from single-celled eukaryotes to vertebrates (Smith, 2009). Depending on the species, they can develop in various host tissues where they form spores that are infective for the next host. Additionally, some microsporidians can be transmitted vertically from the mother to the offspring (Smith, 2009). Microsporidia are frequently found in aquatic systems where the majority of known species infects aquatic invertebrates (Stentiford *et al*., 2013; Stentiford and Dunn, 2014). While there are numerous studies on microsporidians in aquatic crustaceans such as amphipods (Krebes *et al*., 2010; Grabner *et al*., 2015, 2020; Madyarova *et al*., 2015; Weigand *et al*., 2016; Bacela-Spychalska *et al*., 2018; Quiles *et al*., 2019, 2020, 2021), to our knowledge the only microsporidium reported from *Asellus aquaticus* is *Mrazekia argoisi* that was detected in fat body cells of the host (Kudo, 1924).

The freshwater isopod *A. aquaticus* and amphipods of the genus *Gammarus* are both shredders (feeding on larger organic matter, e.g. leaves, and break it into smaller pieces), but the former inhabits predominantly slow-flowing or stagnant waters, while the latter is usually found in faster-flowing waters (Graça *et al*., 1994). *Asellus aquaticus* is the most widespread, and abundant freshwater isopod in Europe and Asia Minor and a recent study shows that it consists of several genetically distinct lineages or operational taxonomic units (OTUs), most of them being potentially distinct (sub-)species that have rather restricted ranges in southern Europe, i.e. on the Balkan Peninsula and the Apennine Peninsula (Sworobowicz *et al*., 2015). The majority of Europe, particularly its central and northern area, is inhabited by the nominative species, *Asellus aquaticus aquaticus*, showing no clear spatio-genetic structure (Sworobowicz *et al*., 2020). Given the fact that microsporidians are common in most groups of crustaceans (Stentiford *et al*., 2013), we would expect a similar microsporidian diversity in isopods as in amphipod hosts. This assumption is supported by the occurrence of other parasite taxa in both groups of crustaceans, e.g. being intermediate hosts for closely related acanthocephalan (Sures, 2014) and trematode parasites (Bock, 1984; Bojko *et al*., 2017). Nevertheless, due to the lack of studies investigating microsporidians in isopods, it is not clear to date whether *A. aquaticus* is an equally suitable host for microsporidians compared to amphipods.

Therefore, the aim of the present study was to obtain a first overview of the presence and diversity of microsporidian parasites in the genetically heterogenous aquatic isopod *A. aquaticus* from a wide geographical range, which also allowed us to investigate potential differences of microsporidian diversity in the genetically distinct host lineages. The presence of microsporidian infections was tested on samples collected previously in the studies of Sworobowicz *et al*. (2015, 2020), including several host OTUs originating from a large geographic area within Europe.

## Materials and methods

### Collection of isopods

Ethanol (99%) fixed individuals of *A. aquaticus* that were collected previously at various localities throughout Europe within the studies of Sworobowicz *et al*. (2015, 2020) were used for the present study (247 individuals from 30 sites in 17 countries, for sampling details see Sworobowicz *et al*., 2015, 2020) (Table 1). In the present study, individuals clustered into OTUs as determined previously by Sworobowicz *et al*. (2015): A (*Asellus aquaticus aquaticus*), D, F and J were used to cover most of the sampling area and the major host OTUs. Depending on the availability of specimens remaining from the study of Sworobowicz *et al*. (2015), 5–10 host individuals were analysed for each sampling site (see Supplemental file 1). Specimens originating from sites where more than 1 MOTU was present were barcoded as described in Sworobowicz *et al*. (2015).

**Table 1.** Details on sampling location and microsporidian prevalence per site in *Asellus aquaticus* hosts.

Location ID	Country	Habitat type	No. of individuals	Longitude	Latitude	No. of infected individuals (Prevalence)
BEL1	Belgium	Pond	5	3.04081	51.17517	0 (0%)
BLR1	Belarus	Stream	10	28.237525	54.6524556	5 (50.0%)
BLR6	Belarus	Spring	10	29.401944	52.03225	0 (0%)
BUL2	Bulgaria	Stream	5	24.162117	43.088867	0 (0%)
EST3	Estonia	Stream	10	26.81035	58.2019	4 (40.0%)
EST6	Estonia	Stream	9	24.7909361	59.1282194	3 (33.3%)
FRA3	France	Pond	7	2.22939	49.92377	3 (42.9%)
FRA6	France	Pond	7	4.25095	49.00859	3 (42.9%)
GBR3	United Kingdom	Stream	10	−1.428568	52.635525	6 (60.0%)
GRE1	Greece	Channel	7	23.6116667	40.6543833	0 (0%)
HUN2	Hungary	Pond	7	18.2614	46.3253	3 (42.9%)
HUN7	Hungary	Pond	7	21.735	47.5003	1 (14.0%)
IRL1	Ireland	Lake	8	−8.332028	52.943331	2 (25.0%)
ITA3	Italy	Channel	10	13.3729	45.7688	3 (30.0%)
ITA14	Italy	Channel	8	10.2989	45.3662	3 (37.5%)
ITA28	Italy	Stream	9	13.0797	42.7949	3 (33.3%)
MKD1	North Macedonia	Lake	5	20.789433	41.110779	1 (20.0%)
MKD2	North Macedonia	Spring	10	20.741956	40.913694	2 (20.0%)
MKD3	North Macedonia	Spring	8	20.75846	40.91487	2 (25.0%)
MNE11	Montenegro	Lake	6	19.2230556	42.2286111	0 (0%)
MNE23	Montenegro	Spring	8	18.950826	42.839135	0 (0%)
MNE32	Montenegro	Spring	10	19.1225	42.4844444	0 (0%)
POL29	Poland	Pond	10	22.22579	49.56936	9 (90.0%)
POL44	Poland	Pond	10	19.16035	51.10418	1 (10.0%)
ROM1	Romania	Lake	8	28.61705	44.2725	1 (12.5%)
ROM2	Romania	Stream	8	20.5838889	46.0375	4 (50.0%)
SVK4	Slovakia	Stream	8	19.240769	48.141169	0 (0%)
SVK7	Slovakia	Stream	7	19.718972	48.295056	6 (85.7%)
TUR1	Turkey	Stream	10	35.1530694	42.0231389	0 (0%)
UKR13	Ukraine	Stream	10	35.046183	48.464717	1 (10.0%)

### Sample processing and molecular detection of microsporidians

To analyse the relationship between host size and parasite infection, the length of each *A. aquaticus* individual was measured according to images taken with a stereo microscope equipped with a camera (moticam 2300, Motic^®^) that was calibrated with a scaled slide. The animals were cut approximately in the sagittal plane and the intestine was removed to avoid contamination with gut content. The DNA was extracted from the remaining tissue following the procedure described in Grabner *et al*. (2015). Detection of microsporidians was conducted using the universal microsporidian primers V1 (5′-CACCAGGTTGATTCTGCCTGAC-3′) (Zhu *et al*., 1993) and mic-uni3R (5′-ATTACCGCGGMTGCTGGCAC-3′) (Weigand *et al*., 2016). PCR thermal profiles and reaction volumes were conducted as described in Weigand *et al*. (2016). PCR products were purified using an E.Z.N.A. Cycle Pure kit (Omega Bio-Tek) and sent for Sanger sequencing (Microsynth Seqlab) using primer V1. Raw sequences were quality-checked using Geneious v2022.0.1 (Biomatters).

### Data analysis

The dependency of habitat type or host MOTU to the frequency of microsporidian-infected *A. aquaticus* was tested by Pearson's *χ*^2^ test (function “chisq.test”) in R v4.1.0 (R Core Team, 2021). Size differences of infected and uninfected host individuals were tested by a 2-sample *t*-test (function “t.test” in R). Graphs were generated in R using the ggplot2-package v3.3.5 (Wickham, 2016).

Microsporidian molecular taxonomic units (MICMOTUs) were identified from the sequences if the genetic similarity between the isolates was less than 96%. Microsporidian prevalence was calculated for each sampling site. The results were depicted in a map using QGIS v3.16.7 (QGIS.org, 2021) and Natural Earth (naturalearthdata.com). Similarity to known sequences was analysed by BLAST-search (https://blast.ncbi.nlm.nih.gov/Blast.cgi).

MEGA X v10.2.6 (Kumar *et al*., 2018) was used to align the consensus sequences of the respective MICMOTUs with the ClustalW algorithm and default parameters. Calculation of p-distances (including transitions and transversions, using gamma-distribution with invariant sites for the substitution rate, partial deletion with 95% site cut-off, and 1000 bootstrap replicates) was also conducted with MEGA X.

The alignment for the phylogenetic analysis included sequences from GenBank that were most similar to the detected microsporidian isolates and representatives of the major microsporidian groups according to the phylogeny by Bojko *et al*. (2022). Sequences were aligned using the MAFFT algorithm v7.48 (Katoh and Standley, 2013) from the EMBL-EBI sequence tools (Madeira *et al*., 2022). Gaps and unaligned regions were removed manually, and the final alignment had a length of 315 bp and contained 153 sequences. Model selection and phylogenetic analysis were conducted with IQ-TREE v1.6.12 (Nguyen *et al*., 2015; Kalyaanamoorthy *et al*., 2017) using ultrafast bootstrap (UFBoot) to test branch support (Hoang *et al*., 2018). *Amphiamblys* sp. (KX214674) and *Chytridiopsis typographi* (MH728789) were used as outgroups. The tree was visualized with the program FigTree v1.4.4 (Rambaut, 2010).

## Results

### Prevalence of microsporidians

In total, 247 individuals of *A. aquaticus* from 30 sites were PCR-tested for microsporidian infections of which 66 were found positive (total prevalence 26.7%, Table 1). At 9 sites, no infected *A. aquaticus*-individual was detected (5–10 specimens investigated). The 2 sites with the highest prevalence were POL29 (90.0%, *n* = 10) and SVK7 (85.7%, *n* = 7) and the sites with the lowest prevalence were UKR13 and POL44 (10.0%, *n* = 10; see Table 1).

The analysis of the frequency of infected individuals and habitat type showed a significant relationship (Pearson's *χ*^2^ test, (*χ*^2^ = 36.044, df = 14, *P* < 0.005). The lowest proportion of infected individuals (8.7%) was found for springs, while the highest proportion was recorded for ponds (37.7%; see Table 2). Furthermore, as significant dependency of the 4 distinct host OTUs (according to Sworobowicz *et al*., 2015) to the frequency of microsporidian-infected individuals was detected (Pearson's *χ*^2^ test, (*χ*^2^ = 15.645, df = 3, *P* < 0.005) (see Supplementary file 1 for raw data). No infections were recorded for OTU F, even though it occurred at 5 sites (BUL2, GRE1, MNE11, 23, 32). The highest proportion of microsporidian infections was found in OTUs J (32.1%) and A (31.7%) followed by OTU D (21.7%) (Table 3).

**Table 2.** Number of *Asellus aquaticus* hosts and proportion of individuals infected by microsporidians for each habitat type

Habitat (no. of sites)	Total no. of hosts	No. of infected hosts	Proportion of infected hosts (%)
Spring (5)	46	4	8.7
Lake (4)	27	4	14.8
Channel (3)	25	6	24.0
Stream (11)	96	32	33.3
Pond (7)	53	20	37.7

**Table 3.** Number of *Asellus aquaticus* hosts and proportion of individuals infected by microsporidians in each host OTU

Host MOTU (no. of sites)	Total no. of hosts	No. of infected hosts	Proportion of infected hosts (%)
A (20)	164	52	31.7
D (3)	22	5	21.7
F (5)	32	0	0
J (4)	28	9	32.1
Total (30)	247	66	26.7

### Relation of host size and infection

**Fig. 1.**
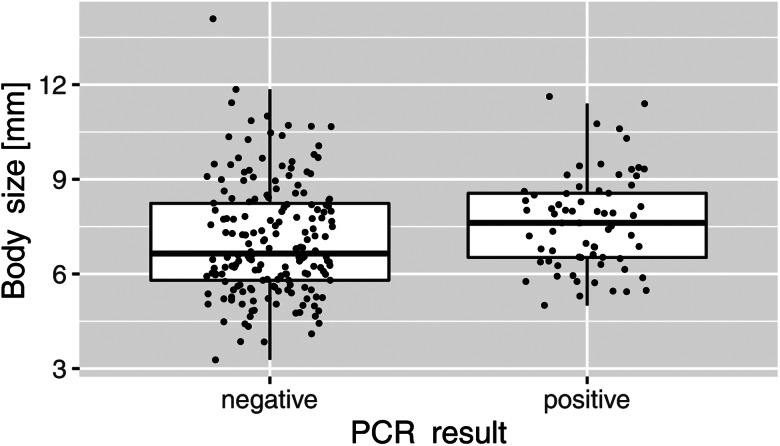
Size dependence of positive (*n* = 66) and negative (*n* = 181) results of the microsporidian PCR. Asterisks indicate significant difference (*P* < 0.05).

A significant difference was found for the size of infected and uninfected *A*. *aquaticus* individuals (*t* = −2.26, *P* < 0.05). Infected individuals were larger than uninfected specimens (mean 7.64 mm *vs* 7.08 mm; Fig. 1; see Supplementary file 1 for individual measurements).

### MICMOTUs and their geographic location

In total, 57 microsporidian sequences were obtained and assigned to 17 different MICMOTUs. No usable sequence could be obtained from 9 individuals due to low-quality reads and high background. The *P*-distances (proportion of nucleotide sites at which 2 sequences being compared are different) between the MICMOTUs were between 0.046 (MICMOTUs 5 and 7) and 0.376 (MICMOTUs 2 and 4). For details on the *P*-distances see Supplementary file 2.

**Fig. 2.**
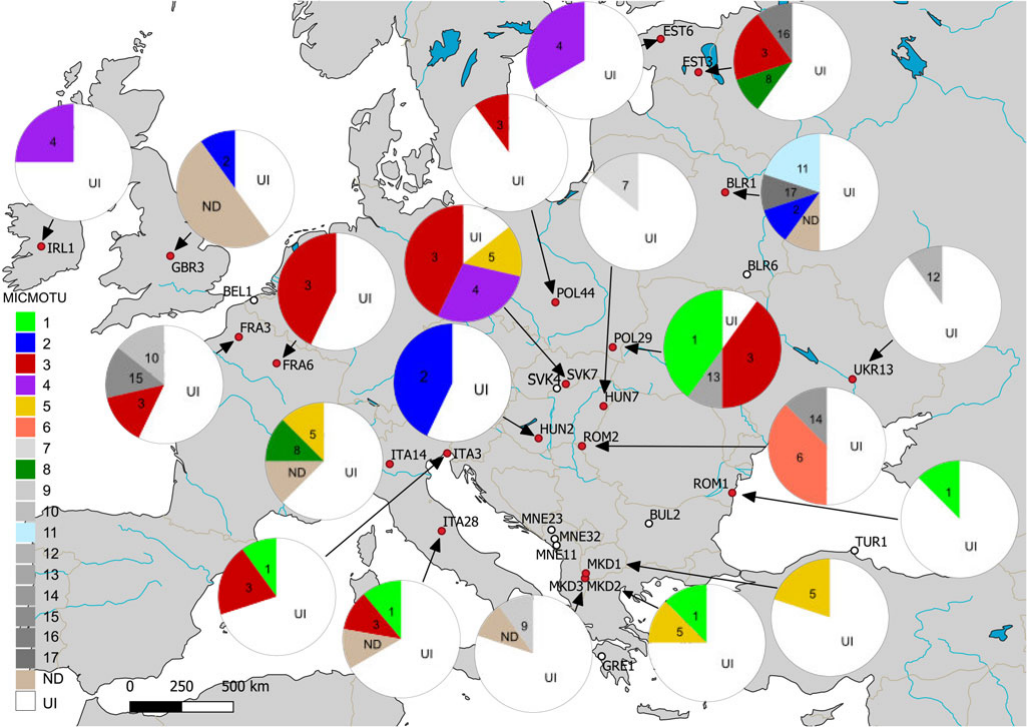
Map showing the sampling locations and pie-charts showing the prevalence of the respective microsporidian MOTUs (MICMOTU) at each site. At sites indicated with white dots, no infections were found. Red dots indicate sites where infected *Asellus aquaticus* were detected. Numbers in pies indicate the respective MICMOTU. UI, uninfected, ND, not determined (PCR positive but sequence was too short or of poor quality). Greyscale fill indicates MICMOTUs that were detected only in a single host individual. Please note the uncertainty of prevalence values given due to low sample size.

MICMOTU 3 was the most common and was revealed from 17 host individuals, followed by MICMOTUs 1 and 4 with 8 host individuals infected by these parasites. MICMOTUs 2 and 5 were represented by 5 and 4 host individuals, respectively. MICMOTUs 6, 7, 8 and 11 were detected in 2–3 isopods, while all other MICMOTUs were single findings. MICMOTUs 1 and 5 occurred in all host OTUs, while MICMOTU 9 was found only in OTU D as a single finding. The most abundant host OTU A harboured all MICMOTUs (except 9). The map in Fig. 2 shows the geographic distribution of the MICMOTUs.

Based on the available dataset, there was no obvious geographical distribution of the MICMOTUs isolated from *A*. *aquaticus*. The most abundant MICMOTU 3 occurred at 8 sites throughout Europe. MICMOTU 1 was located mostly in Southern Europe (Italy, North Macedonia, Romania), but was also found at 1 site in Central Europe (Poland). MICMOTU 4 was found at 2 northern sites (Ireland, Estonia), but also in Slovakia (Fig. 2).

### Phylogenetic reconstruction of MICMOTUs

**Fig. 3.**
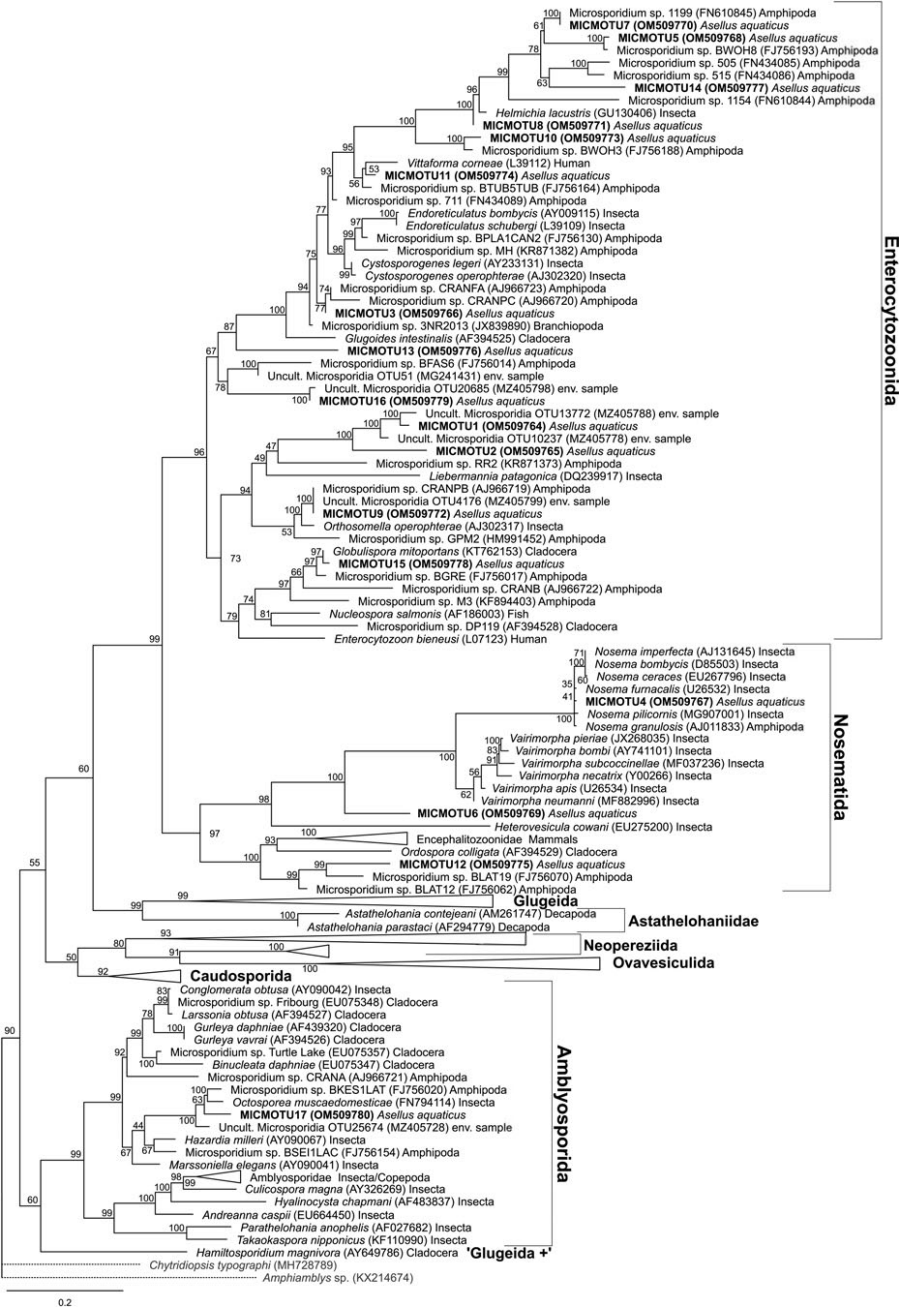
Maximum likelihood phylogenetic tree reconstruction with 307 ultrafast bootstrap iterations of the microsporidian MOTUs (MICMOTUs) detected in *Asellus aquaticus* (in bold) including microsporidian sequences representing the recent microsporidian taxonomy *sensu* Bojko *et al*. (2022). Substitution model was GTR + F + R5. *Amphiamblys* sp. (KX214674) and *Chytridiopsis typographi* (MH728789) were used as outgroups. GenBank accession numbers are shown in brackets and the host group/sample type for each sequence isolate is indicated. Branches that did not contain microsporidians from *A. aquaticus* were collapsed to make the tree clearer. The same tree with branches not collapsed can be seen in Supplementary file 3.

The nucleotide sequence data of the MICMOTUs are available in the GenBank database under the accession numbers OM509764–OM509780 (also shown in Fig. 3). The phylogenetic inference illustrated the phylogenetic position of the microsporidian isolates from *A*. *aquaticus* found in the present study (Fig. 3). Most MICMOTUs clustered in the groups Nosematida and Enterocytozoonida while only MICMOTU 17 was found in the group Amblyosporida (taxonomy according to Bojko *et al*., 2022). MICMOTU4 was located among the *Nosema* spp. (in Nosematida), while MICMOTU6 was located in a position basal to both the *Vairimorpha* and *Nosema* spp. MICMOTU12 also clustered in the Nosematida close to previous isolates from amphipods.

MICMOTU5, 7, 8, 10 and 14 were found in the Enterocytozoonida in a branch dominated by microsporidians detected in amphipods, with MICMOTU 7 being almost identical to Microsporidium sp. 1199 (FN610845) (Fig. 3). The remaining MICMOTUs were located all over the Enterocytozoonida and it is noteworthy that MICMOTUs 1, 2, 9 and 16 were most closely related to different environmental samples of microsporidians (without host record), all found in the study of Dubuffet *et al*. (2021).

## Discussion

The present study provides the first survey data on microsporidian diversity in *A. aquaticus* from a total of 30 sampling sites throughout Europe. A total of 17 MICMOTUs were identified, of which only 5 were detected in 4 or more host individuals. Eight MICMOTUs were only detected in single individuals. For the latter, it is doubtful as to whether these isolates were true infections of *A*. *aquaticus* specimens or rather contaminations by spores or DNA of microsporidians actually infecting other species. Even though the intestines of the hosts were removed prior to DNA extractions, contaminations with remaining of the intestinal content cannot be ruled out completely. Such contaminations with environmental spores, but also co-infections with 2 or more species of microsporidians might explain the failure to obtain sequencing results (high background or short reads) in some cases, indicating low amounts of microsporidian DNA in the sample or mixtures of the DNA of different parasite species. On the other hand, such rare microsporidians were also detected in several species of amphipods and it is assumed that these are true infections (Grabner *et al*., 2015; Grabner, 2016). Particularly the problem of co-infections in the same host individual should be eliminated in follow-up studies by using metabarcoding techniques like those recently applied for microsporidians (Trzebny *et al*., 2020). On the other hand, by removing the intestine, infections in this organ could also have been overlooked. Therefore, the microsporidian diversity in *A. aquaticus* might even be higher.

Compared to studies on other aquatic organisms such as amphipods or insect larvae, where the prevalence rates are often 50% or higher (Grabner *et al*., 2015; Grabner, 2016), the prevalence found in *A. aquaticus* in the present study was lower, and highly variable (0–90%) between the different sampling sites. However, it must be noted that the small sample size (at few sites only 5 individuals were available) increases the uncertainty of the estimate, as a low prevalence would remain undetected. The significantly higher prevalence found in larger hosts might indicate an accumulation of infective spores during the life span of the host or an increased susceptibility of larger hosts due to a switch in feeding habits or microhabitat preference. This pattern does not seem to be universally valid for microsporidia, as in a previous study no weight difference was found between amphipods that were infected or uninfected with the microsporidium *Dictyocoela duebenum* (Chen *et al*., 2015). In contrast, infections with *Nosema* sp. in *Gammarus duebeni* caused a size reduction of infected females (Terry *et al*., 1998). Therefore, the effect of microsporidian infection on host weight/size seems to depend on the respective host–parasite system. In the present study, MICMOTU4 was found to be closely related to *Nosema* spp., indicating that this and maybe other microsporidians in *A. aquaticus* might cause a size reduction of the host. Due to the overall low sample size, we did not differentiate between the different MICMOTUs for the analysis. Therefore, it has to be noted that an analysis of single MICMOTUs using a larger dataset would provide more differentiated results, as the effects of the different microsporidians for their host might be quite different.

There was a significant relationship between the host OTUs, as determined in the study of Sworobowicz *et al*. (2015), and the frequency of infected *A. aquaticus*, even though the proportions of infected individuals was similar for OTUs A, D and J. An exception refers to the host OTU F with zero infections, even though this OTU was represented by 32 *A. aquaticus* individuals, however with most of them originating from 3 sites in Montenegro. This might be due to local factors affecting the host populations (e.g. drought), or seasonal changes in parasite prevalence. In a study on the prevalence of microsporidians in the amphipod *Paracalliope fluviatilis*, prevalence varied over time and no microsporidians were detected at some time points (Park and Poulin, 2021). A similar time course may account for the absence of Microsporidia at the 3 sites in Montenegro, which coincidentally corresponds to the presence of OTU F at this location. Another possible reason for the lack of microsporidians in OTU F might be the relative isolation of the sampling sites in Montenegro (Lake Skadar system) which is characterized by a high rate of endemic species (Grabowski *et al*., 2018). Therefore, OTU F might have lost their microsporidian parasites during the colonization of the system and so far, no microsporidians of *A. aquaticus* were co-introduced to the area.

The associations of host OTUs and frequency of microsporidians in the present study are in contrast to the study by Wilkinson *et al*. (2011), who found little support for coevolution of Microsporidia of the genus *Dictyocoela* with their gammarid hosts. Nevertheless, there is strong evidence for co-diversification of microsporidians and their amphipod hosts (Park *et al*., 2020; Quiles *et al*., 2020). This indicates that the distribution pattern of microsporidians in amphipods is shaped both by ancient host–parasite associations and more recent horizontal transfer between host species or lineages (Quiles *et al*., 2021). The same might be true for *A. aquaticus* and their microsporidians, but it would require a larger sample size and a parasite species-specific analysis to substantiate the link between host OTU and the frequency of infection observed in the present study.

The frequency of infected *A. aquaticus* was significantly related to habitat type, which might be explained by specific habitat characteristics (e.g. temperature, flow velocity, nutrient availability) that can affect infection rate and thereby parasite prevalence (Marcogliese, 2001; Kelly *et al*., 2002; Narr *et al*., 2019). *Asellus aquaticus* collected from spring habitats in the present study showed the lowest proportion of infected individuals, which is in contrast to findings from amphipods, where species-rich microsporidian communities were found in niphargid amphipods from such ground water-dependent habitats (Grabner *et al*., 2020).

The genus *Asellus* is widely distributed throughout Europe (Sket, 1994), therefore a spatially homogenous distribution of associated microsporidian parasites would be expected with unique parasites in remote locations. Nevertheless, the distribution of the different MICMOTUs throughout Europe did not show a conclusive pattern. This is similar for microsporidians of amphipods that show a pan-European distribution without a clear geographic pattern (Krebes *et al*., 2010; Grabner *et al*., 2015; Bacela-Spychalska *et al*., 2018; Prati *et al*., 2022). Furthermore, the rather small sample size has to be taken into account, and it is likely that a higher number of sampling sites and more tested individuals would probably show a more even distribution of most MICMOTUs.

The phylogenetic analysis shows the proximity of MICMOTUs detected in *A. aquaticus* to various branches including microsporidians of amphipods. Some isolates were similar to those detected previously in environmental samples from aquatic habitats and they might originally be parasites of *A. aquaticus* (Dubuffet *et al*., 2021). Interestingly, MICMOTU4 from the present study was closely related to *Nosema* spp., a group of microsporidians that was mostly found to parasitize insects (with the exception of *N. granulosis* from amphipods) (Tokarev *et al*., 2020). This might indicate that the host diversity within the genus *Nosema* and possibly also *Vairimorpha* will extend to other groups of arthropods, in the course of future studies.

Most of the more common MICMOTUs detected in *A. aquaticus* (MICMOTU1, 2, 3, 5) were highly similar to microsporidian isolates from amphipods from the group Enterocytozoonida. It raises the question, if these isopod and amphipod microsporidians are closely related but distinct species, or if the same microsporidian species is a host generalist that is able to infect different groups of aquatic crustaceans. As we know from microsporidians with well-described life cycles, both strategies (host generalists and host specialists) can be found in different microsporidian species (Wadi and Reinke, 2020), but generally low host specificity was found for microsporidians infecting amphipods (Prati *et al*., 2022). In this context, it is interesting to note that no MICMOTUs from *A. aquaticus* were related to microsporidians from the group Glugeida that includes common parasites of amphipods like *Dictyocoela* or *Cucumispora* spp.

## Conclusion

The present study provides a first overview on the microsporidian diversity in different genetic lineages of *A. aquaticus*. Several issues arise from this study that should be addressed in the future: First of all, more host individuals should be analysed to detect MICMOTUs that might indicate coevolution of host and parasite lineages, and to clarify the status of “rare” microsporidians as true infections or contaminations. Furthermore, the geographic distribution of the microsporidians should be studied in closer detail to substantiate the presence (or inferred absence) of common microsporidian species throughout the study area. And finally, the ratio of co-infections of 2 or more microsporidians in the same host should be addressed.

## Supplementary material

For supplementary material accompanying this paper visit https://doi.org/10.1017/S003118202200124X. click here to view supplementary material
