# Supplementary material for: Microsporidian diversity in the aquatic isopod Asellus aquaticus
Source: Parasitology. 2022 Aug 25;149(13):1729–36. doi: 10.1017/S003118202200124X (PMC10090770; doi:10.1017/S003118202200124X)
Supplement: Supplementary file 1 [file S003118202200124Xsup.zip › S003118202200124Xsup003.pdf]

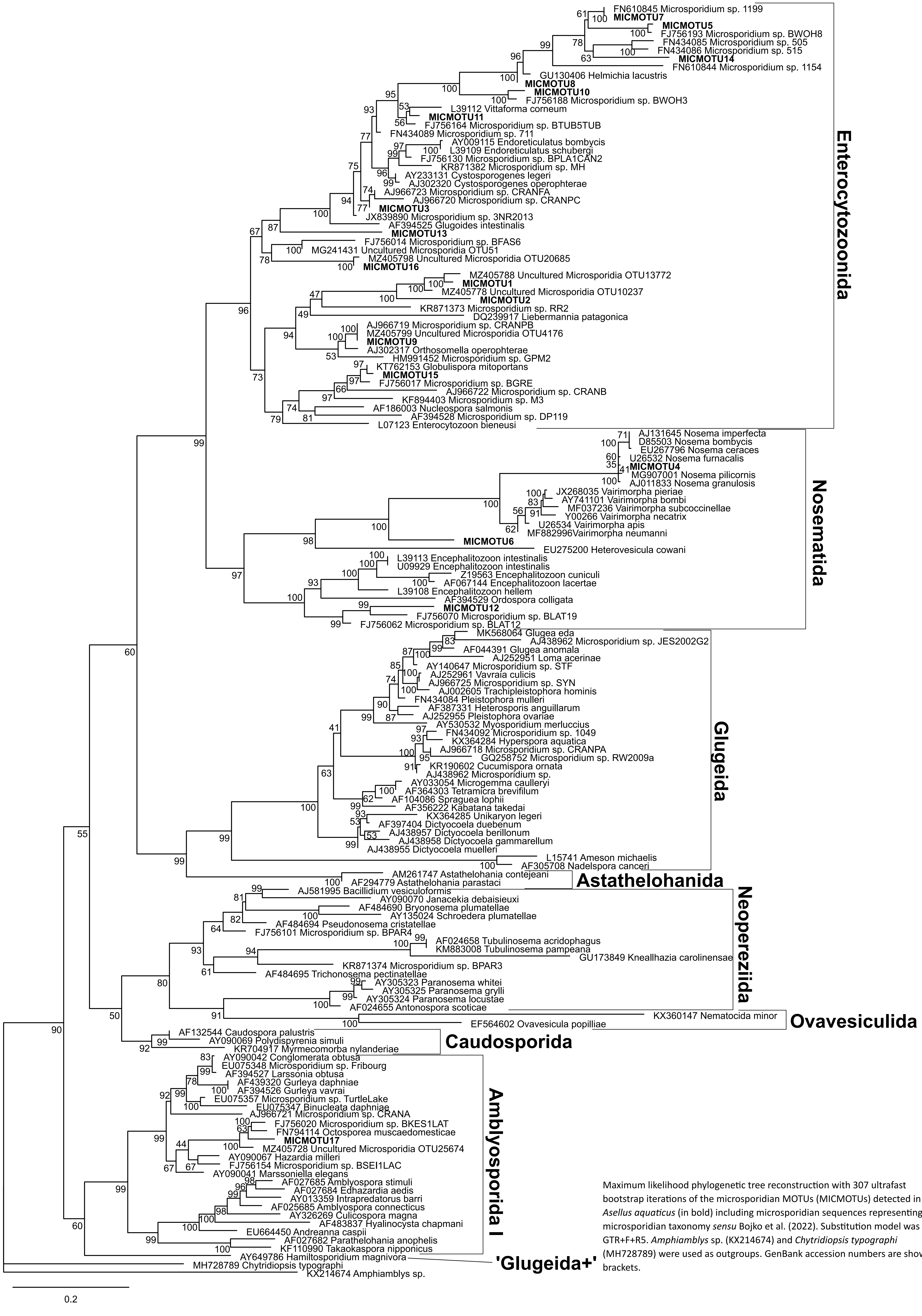


Maximum likelihood phylogenetic tree reconstruction with 307 ultrafast bootstrap iterations of the microsporidian MOTUs (MICMOTUs) detected in *Asellus aquaticus* (in bold) including microsporidian sequences representing the microsporidian taxonomy *sensu* Bojko et al. (2022). Substitution model was GTR+F+R5. *Amphiamblys* sp. (KX214674) and *Chytridiopsis typographi* (MH728789) were used as outgroups. GenBank accession numbers are shown in brackets.
